# The Aplidin analogs PM01215 and PM02781 inhibit angiogenesis *in vitro* and *in vivo*

**DOI:** 10.1186/s12885-015-1729-4

**Published:** 2015-10-19

**Authors:** Bojana Borjan, Normann Steiner, Silvia Karbon, Johann Kern, Andrés Francesch, Martin Hermann, Wolfgang Willenbacher, Eberhard Gunsilius, Gerold Untergasser

**Affiliations:** 1 Department of Internal Medicine V, Innsbruck Medical University, Innrain 66, 6020 Innsbruck, Austria; 2 Oncotyrol GmbH, Karl Kapfererstrasse 5, 6020 Innsbruck, Austria; 3 Pharmamar, R&D Department, Avda de los Reyes 1, 28770 Colmenar Viejo, Madrid Spain; 4 Department of Anesthesiology & Critical Care Medicine, Innsbruck Medical University, Innsbruck, Austria; 5 Tyrolean Cancer Research Institute, 6020 Innsbruck, Austria

**Keywords:** Aplidin analogs, Angiogenesis, Oxidative stress, UPR, Vasohibin, Dickkopf-3, p16^INK4A^

## Abstract

**Background:**

Novel synthesized analogs of Aplidin, PM01215 and PM02781, were tested for antiangiogenic effects on primary human endothelial cells *in vitro* and for inhibition of angiogenesis and tumor growth *in vivo*.

**Methods:**

Antiangiogenic activity of both derivatives was evaluated by real-time cell proliferation, capillary tube formation and vascular endothelial growth factor (VEGF)-induced spheroid sprouting assays. Distribution of endothelial cells in the different phases of the cell cycle was analyzed by flow cytometry. Aplidin analogs were tested*in vivo*in chicken chorioallantoic membrane (CAM) assays.

**Results:**

Both derivatives inhibited angiogenic capacities of human endothelial cells (HUVECs) *in vitro* at low nanomolar concentrations. Antiangiogenic effects of both analogs were observed in the CAM. In addition, growth of human multiple myeloma xenografts*in vivo*in CAM was significantly reduced after application of both analogs. On the molecular level, both derivatives induced cell cycle arrest in G1 phase. This growth arrest of endothelial cells correlated with induction of the cell cycle inhibitor p16^INK4A^ and increased senescence-associated beta galactosidase activity. In addition, Aplidin analogs induced oxidative stress and decreased production of the vascular maturation factors Vasohibin-1 and Dickkopf-3.

**Conclusions:**

From these findings we conclude that both analogs are promising agents for the development of antiangiogenic drugs acting independent on classical inhibition of VEGF signaling.

**Electronic supplementary material:**

The online version of this article (doi:10.1186/s12885-015-1729-4) contains supplementary material, which is available to authorized users.

## Background

Growing tumors undergo an angiogenic switch, i. e. tumor cells start to produce angiogenic growth factors that cause destabilization of existing blood vessels, angiogenic sprouting and generation of new immature blood vessels [1]. Normally, endothelial cells are growth-arrested in the human vascular system and stabilized by mural cell coverage. Upon hypoxia or wound healing, factors like vascular endothelial growth factor (VEGF) and basic fibroblast growth factor (FGF2) induce vascular basement membrane degradation, invasion, migration and proliferation of endothelial cells [2]. After capillary tube formation, endothelial cells recruit new mural cells to cover and stabilize newly formed blood vessels [3]. Growing tumors make use of these mechanisms under hypoxic conditions and generate new blood vessels to enlarge and metastasize [4].

Antiangiogenic therapies in cancer medicine make use of drugs that inhibit proliferation of endothelial cells and induce stabilization and maturation of blood vessels [5]. Due to the fact that tumor blood vessels are leaky and immature, they affect blood flow and interstitial blood pressure [6]. Stabilization of blood vessels ensures better delivery of chemotherapeutic drugs to the tumor and enables interstitial blood pressure to be lowered. Thus, cancer medicine uses antiangiogenic drugs like neutralizing antibodies against VEGF-A or small molecules that inhibit the tyrosine-kinase activity of VEGF receptors [7]. Both attempts lead to inhibition of VEGF signaling, but after prolonged treatment alternative pathways cause resistances and further angiogenic processes and tumor progression to develop [8].

This study analyzed substances that are able to inhibit proliferation of human endothelial cells at low non-toxic nanomolar concentrations, thereby inducing growth arrest in tumor endothelial cells. Optimal antiangiogenic compounds should inhibit the proliferation of tumor endothelial cells, but not induce apoptosis in growth-arrested endothelial cells, such as normal endothelial cells in the vascular system. Both drugs, bortezomib and Aplidin, have been shown to exert potent anti-myeloma activities by inducing apoptosis in multiple myeloma cell lines [9–13]. Apart from this anti-myeloma activity both display antiangiogenic activity *in vitro* and *in vivo* in different tumor models independent of inhibition of VEGF signaling [9, 11, 13–15]. More than 200 different Aplidin analogs were synthesized and screened for cytotoxic activities against cancer cell lines (WO 02002596). Here, we identified and characterized two novel analogs with reduced *in vitro* cytotoxicity on human primary cells and more easy chemical synthesis than Aplidin™ and tested them in comparison to the established drugs bortezomib and Aplidin^TM^ for their antiangiogenic effects.

## Methods

### Substances

Bortezomib was purchased from Selleckchem and dissolved in DMSO (SIGMA Biochemicals) to a stock solution of 250 mM. Aplidin™ and Aplidin analogs PM01215 and PM02781 were synthesized in Pharmamar and dissolved in DMSO to stock solutions of 250 mM and stored in aliquots at −80 °C. All stocks were further diluted with DMSO to working concentrations of 1 mM and stored at −20 °C. N-acetyl cysteine (NAC, Sigma Biochemicals) was dissolved in distilled sterile water, and 30 % H_2_O_2_ was purchased from Merck. Thapsigargin was purchased from Life Technologies and dissolved in DMSO (SIGMA Biochemicals) to a stock solution of 1 mM.

### Cell culture

Human endothelial cells from different donors (HUVECs, *n* = 3) were purchased from PromoCell after immunohistochemical testing (vWF+, CD31+, ASMA-). HMECs were cultivated in endothelial cell growth medium (EGM2) with recommended supplements (PromoCell) on collagen type I (Sigma Biochemicals) -coated ventilated plastic flasks. Cells were passaged using the DetachKit (PromoCell) consisting of 30 mM HEPES, 0.04 %/0.03 % trypsin/EDTA solution and trypsin-neutralizing solution (TNS).

Human mesenchymal cells from bone marrow of various donors (*n* = 3) were purchased from PromoCell after their analysis by flow cytometry (CD31+, CD44+, CD45-, CD105+). Cells were cultivated in RPMI1640 medium (Sigma Biochemicals) with 10 % bovine calf serum (Hyclone) and 100 IU/mL penicillin, 100 μg/mL streptomycin and 2 mM glutamine (all PAA Laboratories GmbH) on uncoated plastic material.

Human PBMNCs from healthy donors (*n* = 3,) were prepared as described elsewhere [16]. In brief, blood samples from blood donors were collected in anticoagulant (EDTA) tubes and transferred to Leucosep® tubes (Greiner Bio-One) containing Ficoll (LSM1077 Lymphocyte separation medium, PAA) for density gradient centrifugation. Thereafter, mononuclear cell faction was washed in PBS, characterized by flow cytometry (size, granularity, CD45+ expression) and used for experiments.

All primary cells were characterized by flow cytometry using a panel of cell type-specific markers (Additional file 1: Table S1) and were tested for the absence of HIV1/2, HBV, HCV and mycoplasma. Only cells of low passages were used for experiments. OPM-2 multiple myeloma cells (AC55) were purchased 2012 directly from DSMZ (Germany), authenticated by us (STR-profiling, flow cytometry: CD138+/CD38+) and cultivated in RPMI1640 medium (Sigma Biochemicals) with 10 % bovine calf serum (Hyclone) and 100 IU/mL penicillin, 100 μg/mL streptomycin and 2 mM glutamine (all PAA Laboratories GmbH) on uncoated plastic material. OPM-2 cells were lentivirally transfected to express eGFP and propagated in the presence of blasticidin (2.5 μg/mL, Invitrogen) before usage for *in vivo* experiments.

### Western Blot analysis

Cells were harvested and lysed in a RIPA buffer (Cell Signaling) containing protease inhibitors (Complete Mini EDTA-free; Roche Applied Science). Total protein (20 μg) was denatured, separated with 4 -20 % SDS-PAGE (Criterion TGX, Bio-Rad) and transferred to an Immuno-Blot™ polyvinylidene difluoride (PVDF) membrane (Bio-Rad). After blocking the membrane in 5 % non-fat milk powder dissolved in phosphate-buffered saline (PBS), membranes were incubated overnight in 3 % non-fat milk powder or 5 % BSA at 4 °C with primary antibodies. Afterwards, membranes were incubated with an HRP-conjugated secondary antibody (Dako Cytomation) diluted 1:2,500. After washing, a chemoluminescent substrate (LumiGLO Reagent and Peroxide, Cell Signaling Technology) was added to the membrane, which was then exposed in the Chemidoc XRS station (Bio-Rad Laboratories). Antibodies used for Western Blot analysis were alpha tubulin (clone B5-1-2; Sigma Biochemicals), p27^Kip1^ (clone G173-524, BD Pharmingen) and p53 (clone PAb1801, Calbiochem), p21^Cip1^ (BD Biosciences), p16^INK4A^ (BD Biosciences), VEGF-R2 (Calbiochem), vasohibin (R&D Systems, Clone 411208), GRP78/HSPA5 (R&D Systems, Clone 474421), and XPB1 (SCBT, M-186). Mouse-anti JNK (Santa Cruz Biotechnology, clone D-2), rabbit anti-phospho-p44/42 MAPK (Erk1/2 D13.14.4E), mouse anti-P44/42 MAPK (Erk1/2, clone L34F12), rabbit anti-phospho-Akt (Ser473), rabbit anti-Akt (pan) and rabbit-anti-phospho-JNK (all purchased from Cell Signaling).

### DKK-3 ELISA

Cells were treated for 72 h with 10 nM solution of the respective compounds. For quantitative measurement of DKK-3 in supernatants a commercially available ELISA (human DKK-3 DuoSet; DY1118, R & D Systems) was used according to the manufacturer’s guidelines.

### Immunofluorescence and confocal microscopy

Cells were seeded on collagen-coated eight-well culture slides (Falcon BD Labware) and incubated with 10 nM of Aplidin, PM01215 and PM02781 for 5 h. Living cells were stained with CellRox®Green reagent to monitor intracellular oxidative stress, and nuclei were stained with NucBlue (Molecular Probes, Life Technologies) according to the manufacturer’s protocol. Confocal microscopy was performed with a spinning disc confocal microscopic system (Ultra VIEW VoX; Perkin Elmer, Waltham, MA, USA) that was connected to a Zeiss AxioObserver Z1 inverted microscope (Zeiss). Images were acquired with Velocity software (Perkin Elmer) using a 63x oil immersion objective with a numerical aperture of 1.42.

### Flow cytometry

Cell death was evaluated by human FITC-labeled Annexin V (Enzo^®^) and 7-amino-actinomycin D (7-AAD, Beckman Coulter) staining. Therefore, cells were resuspended in 200 μL Annexin V Binding Buffer (Abcam) with 2 μL Annexin V and 2 μL PI (20 μg/mL), incubated for 15 min, washed and resuspended in PBS/ 5 % FCS prior to analysis. Cells were examined in the FACSCalibur (Becton-Dickinson, Heidelberg, Germany). Cell cycle analysis was performed with the Coulter DNA PREP Reagent kit (Beckman Coulter).

Primary cells were all characterized by staining with a panel of antibodies and flow cytometric analysis (Additional file 1: Table S1). Therefore, we used anti-human EpCAM/TROP1 Phycoerythrin MAb (Clone 158206), anti-human CD31/PECAM-1 APC MAb (Clone 9G11), anti-human VEGF R2/KDR Phycoerythrin MAb (Clone 89106), anti-human CD45 PerCP MAb (Clone 2D1) and anti-human CD14 Fluorescein MAb (Clone 134620, all from R&D systems).

### Quantitative RT-PCR analysis

Total RNA was isolated from HUVECs using TRI Reagent (Sigma -Aldrich), according to the manufacturer’s instructions. RNA was purified by cell lyses and nucleic acid extraction using the RNeasy Kit (Qiagen). Thereafter, genomic DNA in the RNA samples was digested with the RQ1 DNAse (Promega). The cDNA was amplified from 1 μg total RNA using the SuperScript II Reverse Transcriptase Kit (Invitrogen Life Technologies). For validation, real time RT-PCR was performed using a SensiMix SYBR No-ROX Kit (Bioline) and a Rotor-Gene 6000 detection system (Corbett Research). Primers were designed to amplify specific GAPDH (for: 5-ctgacctgccgtctagaaaa; rev: 5-gagcttgacaaagtggtcgt), TATA Box Binding Protein (for: 5-ggagccaagagtgaagaaca; rev: 5-agcacaaggccttctaacct), DKK3 (for: 5- tcatcacctgggagctagag, rev: 5-caacttcatactcatcgggg); VASH1 (for: 5-agatccccataccgagtgtg, rev: 5-gggcctctttggtcatttcc), p16^INK4A^ (for 5-caacgcaccgaatagttacg, rev: 5-agcaccaccagcgtgtc), p27^KIP1^ (for 5-gccctccccagtctctctta, rev: 5-tcaaaactcccaagcacctc), TP53 (for 5-gttccgagagctgaatgagg, rev: 5-ttatggcgggaggtagactg), X-Box Binding Protein 1 XBP1u (for: 5- agtccgcagcactcagac; rev: 5-gaactgggtccttctgggtag) XBP1s (for: 5- agtccgcagcaggtgcaggc; rev: 5-gaactgggtccttctgggtag), HSP5A (for: 5-ctcgactcgaattccaaaga; rev: 5-aaggggacatacatcaagca), and DDIT3 (CHOP) gene (for: 5-cctcctggaaatgaagaggaaga; rev: 5-tcctggttctcccttggtct).

### Real time cell proliferation assays

Real time cell proliferation experiments were performed using the RTCA DP instrument (Roche Diagnostics GmbH), which was placed in a humidified incubator maintained at 5 % CO_2_ and 37 °C. For proliferation assays, cells were seeded in complete medium in 16-well plates (E-plate 16, Roche Diagnostics GmbH) at a density of 2000 cells/well after coating with 10 μg/mm^2^ fibronectin (Sigma Biochemicals). The plate containing gold microelectrodes on its bottom was monitored every 10 min for 4 h (adhesion process), then once every 30 min, until the end of experiment, for a total of 72 h. Data analysis was performed using RTCA software 1.2 supplied with the instrument.

### Capillary tube formation and angiogenic sprouting assays

Cells were incubated for 12 h with 10 nM of the respective compounds. To analyze tube formation, 24-well plates were coated with 200 μL growth factor-reduced matrigel (BD Biosciences). HUVECs were resuspended in 200 μL EGM-2 medium (1 × 10^5^ cells) containing 10 nM of the respective compound and placed on top of the polymerized matrix; tube formation was observed after 6 hours. Tubes were viewed under an inverted transmission microscope (Zeiss Axiovert 200 M) and documented with a digital imaging system (Axiovision Software, Zeiss).

For sprouting assays HUVEC spheroids were generated overnight in hanging-drop culture consisting of 400 cells in EBM-2 medium, 2 % FCS and 20 % methylcellulose (Sigma Biochemicals). Spheroids were embedded in collagen type I from rat tail (Becton Dickinson) and stimulated with 50 ng/ml VEGF (Sigma Biochemicals) in the presence or absence of compounds or control substances (DMSO, bortezomib). Sprouts were also analyzed by inverted transmission-microscopy (Zeiss Axiovert 200 M) and documented by a digital imaging (Axiovision Software, Zeiss). The cumulative sprout length (CSL) was analyzed after printing of high quality pictures and counting by two independent blinded observers.

### Chicken chorioallantoic membrane (CAM)

Fertilized chicken eggs (Gallus domesticus, Charles River) were placed in a 75–80 % humidified 37 °C incubator (Grumbach) to allow normal embryo development. On day three eggs were opened, egg shells removed and embryos were placed in a sterile Petri dish in an egg incubator to induce CAM development. On day 8, when CAM and its vasculature were well developed, all experiments were performed. Subsequently, two rings per chicken were grafted on the CAM. Drugs (10 nmol/ring) with VEGF (1 μg/ring) or drugs alone were applied every second day at the center of Permanox™ rings.

On day 6 post-grafting chicken embryos were sacrificed by hypothermia, blood vessels in the ring area were photographed by stereo microscope (Olympus SZW 10) and vessel density was determined by counting with Photoshop CS4 (Adobe).

### Human tumor xenograft model in the CAM

OPM-2^eGFP^ multiple myeloma cells (2.5 × 10^5^) were mixed with rat-tail collagen and human mesenchymal stromal cells (0.5 × 10^5^) and the 1 nmol of the respective compounds. Collagen drops (30 μl) were placed on parafilm for 30 min to allow polymerization of the extracellular matrix at 37 °C. Then onplants were transferred to the CAM of 7-day-old chick embryos. After 5 days of *in vivo* growth, onplants were documented by the Olympus SZX10 stereomicroscope (Olympus) equipped with an Olympus DFPL 2-4x objective lens connected with a digital camera (Olympus E410) and flexible cold light (KL200; Olympus). Excised xenografts were transferred into 0.5 ml RIPA Buffer (Sigma Aldrich, Linz, Austria) and homogenized with an Ultra Turrax homogenizer three times for 5 s on ice. Thereafter, homogenate underwent three freezing/thawing-cycles in liquid nitrogen and 37 °C water bath. After centrifugation, supernatants were diluted in assay buffer. GFP levels were measured by Cell Biolabs’ GFP ELISA Kit (San Diego, CA, USA), using biotinylated anti-GFP antibodies, according to the manufacturer’s protocol.

### Senescence-associated beta galactosidase (*SA*-*β*-*gal*) activity assay

Cells were fixed (2 % formaldehyde, 0.2 % glutaraldehyde in PBS) for 5 min at room temperature and rinsed several times in PBS. To measure *SA-β-gal* activity, cells were incubated in a staining solution (4.2 mM citric acid, 12.5 mM sodium phosphate, 158 mM sodium chloride, 0.21 mM magnesium chloride, 2.21 mg/ml potassium ferrocyanide, 1.68 mg/ml potassium ferricyanide, 1 mg/ml X-Gal, pH 6.0) at 37 °C for 24 h. Cells were washed and embedded in PBS, viewed in an inverted transmission microscope and photographed (Zeiss Axiovert 200, Axiovision software).

### Statistical analysis

Statistical analyses were performed with the GraphPad Prism™ software for Windows. Unpaired *t*-test was used to study differences between the means of one treatment group and control. The average scores across treated groups were not compared. Statistical analyses of quantitative PCR data were performed according to the delta Ct method described by Pfaffl et al. [17].

## Results

### PM01215 and PM02781 inhibit cell proliferation and induce cell cycle arrest in human endothelial cells

**Fig. 1:**
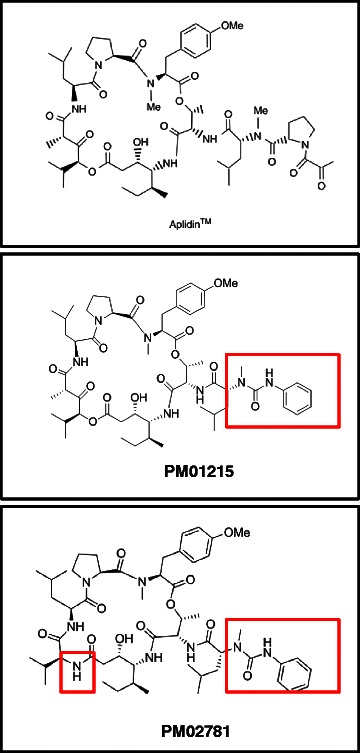
Chemical structure of Aplidin™ and the two novel Aplidin derivatives with modified side-chains (boxes). PM01215 and PM02781 are analogs of Aplidin™, in which the pyruvyl-proline side-chain was replaced with a urea derivative based on phenylisocyanate. Additionally, in PM02781 the α − (α − hydroxyisovaleryl) propionyl (Hip) group that is present in the depsipeptide cycle and connected to an isostatine unit by an ester bond was replaced with L-valine. Note: Both analogs are easier to synthesize than Aplidin™

Testing more than 200 different analogs by Pharmamar in direct comparison to the original compound on tumor cell lines (Patent WO 2002002596) revealed two compounds with similar *in vitro* activity and more easy chemical synthesis than Aplidin™ (Fig. 1).

**Fig. 2:**
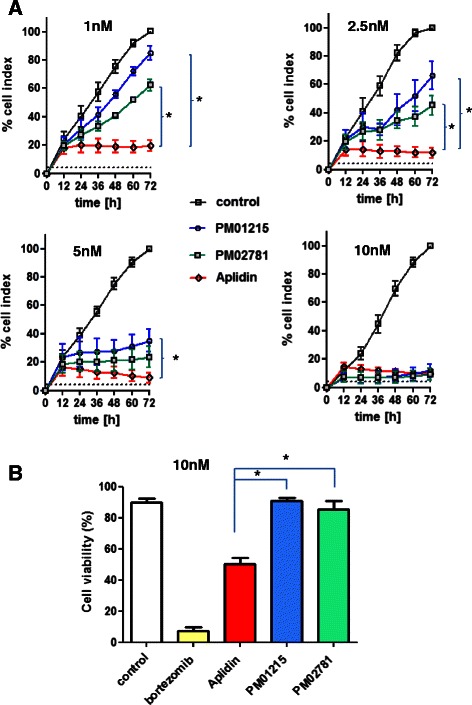
Real-time measurement of endothelial cell proliferation and apoptosis. **a** Proliferation of HUVECs was analyzed in triplicates over a time window of 72 h (*n* = 3, mean ± SEM of three different donors) in direct comparison to respective controls (0.1 % DMSO, control indicates 100 % cell index proliferation on fibronectin-coated E plates). Differences between the means of one treatment group (PM01215 or PM02781) and Aplidin were analyzed at concentrations of 1, 2.5, 5 and 10 nM. Both Aplidin derivates were significantly less potent in inhibition of cell growth when used at 1 and 2.5 nM. At 10 nM both got equally potent like Aplidin. **b** Induction of apoptosis was analyzed after stimulation of human endothelial cells with 10 nM bortezomib, Aplidin, PM01215 and PM02781 for 72 h. Cells were analyzed for cell viability (Annexin V ^negative^, 7-AAD ^negative^) by flow cytometry. In comparison to Aplidin, both analogs were significantly less toxic and induced no apoptosis in human endothelial cells

PM01215 and PM02781 were tested on HUVECs (*n* = 3) in the real-time proliferation system (xCELLigence, Roche Diagnostics) for effects on proliferation and cell numbers. As expected, incubation with 1 nM Aplidin™ completely stopped cell proliferation and 5 and 10 nM already induced apoptosis (Fig. 2a). Both Aplidin analogs showed less inhibition of cell proliferation after 3 days when tested in direct comparison to Aplidin at 1 nM (19.6 ÷.6.5 % cell index for Aplidin™ versus 84.8 ÷ 8.4 % for PM01215 or 62.3 ÷ 7.1 % for PM02781). Concentrations of 10 nM displayed similar activities on inhibition of cell growth after 3 days as the original compound Aplidin™ (10.0 ÷.2.7 % cell index for Aplidin™ versus 17.4 ÷ 4.9 % for PM01215 or 15.6 ÷ 2.2 % for PM02781). In comparison to both analogs, 10 nM Aplidin already induced visible signs of apoptosis and detachment of cells after 72 h.

With regard to induction of apoptosis on human primary endothelial cells, both new Aplidin analogs are less toxic than the original Aplidin™ (Table 1 and Fig. 2b) when used at 10 nM for 72 h (50.3 ÷.6.7 % cell viability for Aplidin™ versus 89.3 ÷ 3.0 % for PM01215 or 85.3 ÷ 6.3 % for PM02781).

Cell cycle analysis by staining with propidium iodide revealed that Aplidin derivatives (10 nM) induced arrest of endothelial cells in G1 phase (Table 2). In comparison to untreated cells the S phase fraction of cells (14.9 ± 1.4 %) was significantly reduced after PM01215 (3.7 ± 1.0 %) and PM02781 (3.4 ± 0.8 %) treatment. Simultaneously, the G1 phase fraction increased from 42.3 ± 2.5 % (control) to 61.1 ± 1.7 % (PM01215) or 61.8 ± 1.0 % (PM02781).

**Table 1:** Apoptosis induced by bortezomib, Aplidin and the Aplidin derivatives PM01215 and PM02781; apoptotic cells were determined by Annexin V ^positive^ staining and flow cytometric analysis; results are displayed as % of apoptotic cells (mean ± SEM) on three different donors 24 h after incubation with the respective compounds

HUVEC (*n* = 3)	Untreated 11.0 ± 0.6				
concentration [nM]		bortezomib	Aplidin™	PM01215	PM 02781
10		45.0 ± 14.0*	10.7 ± 1.3	10.3 ± 0.9	11.0 ± 0.6
50		68.0 ± 11.2*	22.7 ± 2.6*	12.0 ± 0.0	12.7 ± 0.9
100		73.0 ± 10.8*	34.3 ± 2.7*	17.3 ± 0.3*	23.0 ± 2.6*
200		78.3 ± 11.5*	56.0 ± 4.5*	31.0 ± 4.0*	44.3 ± 10. 7*
HDFs (*n* = 3)	Untreated 5.3 ± 0.3				
concentration [nM]		bortezomib	Aplidin™	PM01215	PM 02781
10		4.7 ± 0.3	4.3 ± 0.3	4.7 ± 0.7	4.7 ± 0.3
50		7.7 ± 0.7	5.3 ± 0.7	4.3 ± 0.3	4.7 ± 0.3
100		10.3 ± 0.7*	9.0 ± 0.4	4.3 ± 0.3	6.3 ± 0.3
200		12.7 ± 1.7*	12.3 ± 0.7*	6.0 ± 1.0	7.3 ± 1.2
PBMNC (n = 3)	Untreated 11.3 ± 0.9				
concentration [nM]		bortezomib	Aplidin™	PM01215	PM 02781
10		9.7 ± 2.3	10.3 ± 1.2	9.7 ± 0.9	10.0 ± 1.5
50		25.0 ± 1.0*	11.7 ± 1.3	9.7 ± 1.3	10.0 ± 1.2
100		28.0 ± 2.0*	12.3 ± 1.7	9.7 ± 1.3	11.7 ± 2.3
200		29.7 ± 2.3*	19.3 ± 1.7*	11.0 ± 1.5	12.7 ± 1.9

Primary cells were human umbilical vein endothelial cells (HUVECs), human diploid fibroblasts from bone marrow (HDFs) and human peripheral blood mononuclear cells (PBMNCs). *Indicates *p* value < 0.05

**Table 2:** Cell cycle profiles; DNA content was determined by propidium iodide staining of fixed cells and flow cytometric analysis; results are displayed as % of cells (mean ± SEM) on three different donors 24 h after incubation with a 10 nM concentration of PM01215 and PM02781

	G1 phase	S phase	G2 phase
control	42.3 ± 2.5	14.9 ± 1.4	40.3 ± 1.4
PM01215	61.1 ± 1.7*	3.7 ± 1.0*	34.1 ± 2.4*
PM 02781	61.8 ± 1.0*	3.4 ± 0.8*	33.5 ± 1.7*

*Indicate *p* values < 0.05

### PM01215 and PM02781 induce oxidative stress and terminal growth arrest

**Fig. 3:**
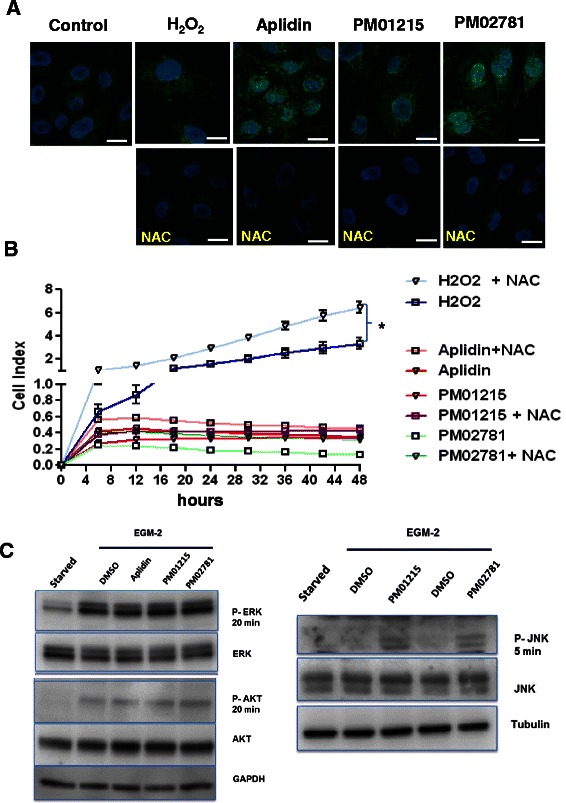
Aplidin analogs induce oxidative stress and activate JNK in human endothelial cells. **a** Generation of reactive oxygen species (ROS) was analyzed by CellRox®Green, a novel fluorogenic probe for measuring oxidative stress in living cells. The cell-permeant dye is weakly fluorescent while in a reduced state and exhibits bright green photostable fluorescence upon oxidation by ROS. Aplidin™ (5nM) and Aplidin derivatives (10 nM) were added to endothelial cells with or without the antioxidant N-acetyl-cysteine (NAC), and ROS in mitochondria monitored (green signals) after 6 h. H_2_0_2_ served as positive control (30 μM, 2 h of incubation). Nuclei were stained with NucBlue. Bars indicate 10 μm. **b** *In vitro* proliferation in presence of antioxidative N-acetyl-cysteine (NAC) was analyzed by real-time proliferation in xCELLigence system. Endothelial cells were pre-incubated for 1 h with antioxidative NAC (25 μM) or medium alone and then stimulated with H_2_0_2_, Aplidin™ (5nM) or Aplidin derivatives (PM01215, PM02781; 10 nM each). In comparison to H_2_0_2_-treated cells, Aplidin analog-treated cells could not be rescued by NAC, indicating a terminal growth arrest. **c** HUVECs were starved overnight and preincubated with drugs for 60 min, then stimulated with standard culture medium supplemented with VEGF, bFGF (100 ng/mL each) and 10 nM of each drug or DMSO (0.1 %) as control. Cytosolic extracts were analyzed by Western Blot for phosphorylation of ERK and AKT after 20 min. Phosphorylation of c-Jun N-terminal kinases (JNK) was analyzed in starved endothelial cells treated with PM01215 and PM02781 for 5 min. In comparison to DMSO-treated cells, Aplidin analogs increased JNK phosphorylation. GAPDH and tubulin served as internal controls for loading and equal protein transfer

Aplidin™ has been reported to induce cell death by oxidative stress [10]. Therefore, we tested both Aplidin analogs with CellRox®Green, a fluorogenic probe for measuring oxidative stress in living cells. The cell-permeant dye is weakly fluorescent while in a reduced state and exhibits bright green photostable fluorescence upon oxidation with reactive oxygen species (ROS). Aplidin derivatives induced ROS already 5 h after incubation. ROS was effectively blocked by adding 25 μM N-acetyl-cysteine (NAC) as antioxidant to the culture medium (Fig. 3a). Furthermore, we analyzed them in a real-time proliferation system in direct comparison to H_2_O_2_, a known inductor of ROS, and attempted to rescue cells by adding NAC to culture medium (Fig. 3b). Growth of endothelial cells was inhibited by 20–30 μM H_2_O_2_. Proliferation of H_2_O_2_-treated cells was significantly increased by adding NAC (25 μM) to the culture supernatant. Aplidin analog-treated cells (10 nM each) could not be stimulated for proliferation, even after incubation with the antioxidant NAC, indicating a terminal growth arrest.

### PM01215 and PM02781 increase phosphorylation of c-Jun N-terminal Kinase (JNK) after mitotic stress

Aplidin analogs were tested for direct effects on phosphorylation of stress (JNK) and mitogenic (ERK) and survival kinases (AKT). In comparison to DMSO–treated endothelial cells, Aplidin analog–treated cells (each 10 nM, *n* = 3) showed no significantly altered phosphorylation of prosurvival kinases upon stimulation with EGM2 medium containing mitogenic growth factors such as VEGF and bFGF. Aplidin analogs did not increase phosphorylation of ERK 20 min after mitogenic stimulation (Fig. 3c). The pro-survival AKT (Protein Kinase-B) was not affected after treatment of endothelial cells with PM01215 and PM02781. In comparison to DMSO treated cells, PM01215 and PM02781 were significantly increasing JNK-phosphorylation in endothelial cells 5 min after mitogenic stimulation (Fig. 3c).

### PM01215 and PM02781 induce premature senescence and expression of the cell cycle inhibitor *p16*^*INK4A*^

**Fig. 4:**
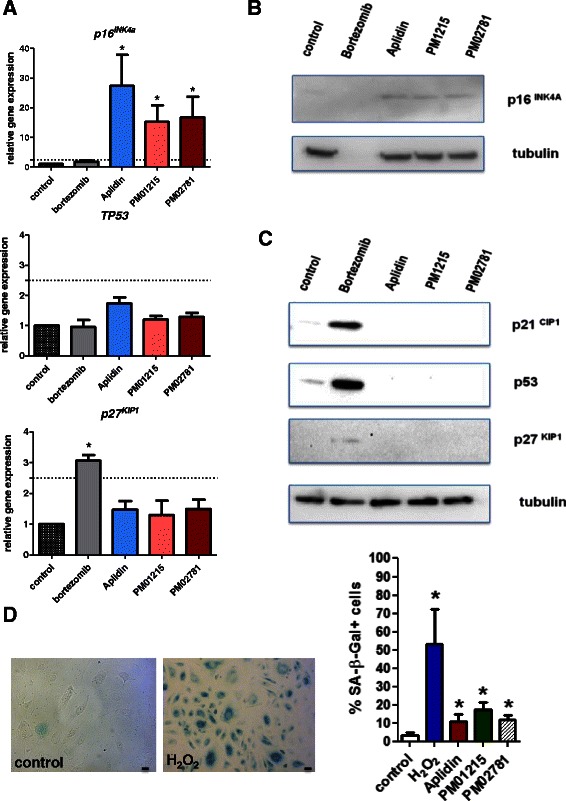
Aplidin analogs induce premature senescence by induction of the cell cycle inhibitor p16^INK4A^. **a** HUVECs (*n* = 3) were stimulated for 24 h with the compounds, after which total RNA and protein was extracted. Gene expression was analyzed by real-time PCR and protein expression by SDS-PAGE and Western Blot. Aplidin™ (5 nM) and Aplidin derivatives (10 nM) induced *p16*^*INK4A*^ gene expression. *TP53* gene expression was induced neither by bortezomib (5 nM) nor by Aplidin (5 nM) or derivatives (10 nM). *P27*^*KIP1*^ gene expression was induced only by bortezomib. **b** Western Blot analysis of p16^INK4A^ protein after treatment with bortezomib, Aplidin™ and derivatives (72 h). **c** Upregulation of p53 and cell cycle inhibitors p21 and p27 after treatment with the proteasome inhibitor bortezomib (24 h). Aplidin™ and derivatives did not induce p53, p21 or p27 proteins, even after 72 h of incubation (data not shown). Tubulin alpha served as loading control. **d** Staining for senescence-associated beta galactosidase in human endothelial cells treated for 72 h with Aplidin™ (5 nM) and analogs (10 nM each). In comparison to control Aplidin analog-treated cells show an increased number of blue (SA-β- gal ^positive^) cells, like the positive control H_2_O_2_. Differences between the means of one treatment group and untreated control were analyzed. The level of significance for the analysis was set at *p* < 0.05. Bars indicate 20 μm

Aplidin analogs induced growth arrest in endothelial cells by induction of *p16*^*INK4A*^ gene expression (Fig. 4a) already 24 h after incubation. Bortezomib (5 nM) did not affect *p16*^*INK4A*^ gene levels. Western Blot analysis after 72 h confirmed that p16 protein was induced by Aplidin and analogs, whereas bortezomib-treated cells were already apoptotic and showed degradation of cellular protein (Fig. 4b). Bortezomib significantly elevated *p27*^*Kip1*^ gene expression, but had no effect on *TP53* gene expression 24 h after stimulation (Fig. 4a). Aplidin analogs did not affect *TP53* or *p27*^*Kip1*^ gene expression (Fig. 4a). As expected from its proteasome inhibitory property, bortezomib (5 nM) increased p53 and p21 protein in endothelial cells after 24 h of incubation (Fig. 4c). Aplidin analogs did not affect p53, p21 or p27 protein levels 24 h after incubation (Fig. 4c) or 72 h after stimulation (data not shown).

Since oxidative stress brings on premature senescence in primary cells, we analyzed the effects of PM01215 and PM02781 for induction of growth arrest by staining for senescence-associated β-galactosidase activity (SA-β-gal) after three days of incubation. In comparison to untreated control cells, Aplidin analogs (10 nM each) significantly increased the number of SA- β -gal positive cells (Fig. 4d).

### Aplidin and analogs do not induce canonical unfolded protein response

**Fig. 5:**
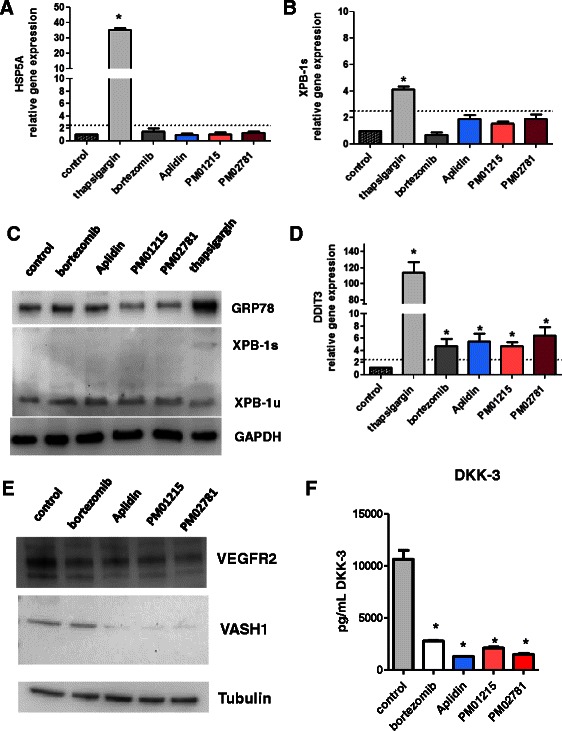
Aplidin and analogs do not induce canonical unfolded protein response but downregulate vascular maturation factors *VASH1* and *DKK3*. Three different branches of UPR, the master regulator GRP78/HSP5A, the IRE1a/XPB-1 s and PERK/CHOP/DDIT3 pathway were analyzed. In contrast to thapsigargin (10 nM), bortezomib (5 nM), Aplidin (5 nM) and analogs (10 nM) did not induce HSP5A gene expression (**a**) and splicing XPB1 within 24 h (**b**). **c** Only thapsigargin was able to increase GRP78 and XPB1 s protein as analyzed after 72 h by Western Blot. **d** DDIT3/CHOP was significant elevated on gene expression after 24 h in bortezomib, Aplidin and analog-treated cells. Aplidin and derivates did not display such an inductive response as observed for thapsigargin. **e** For analysis of vascular maturation factors HUVECs were incubated with bortezomib (5nM), Aplidin™ (5nM) or Aplidin analogs (each 10 nM) Western Blot analysis of VASH1 protein in cytosolic extracts of bortezomib, Aplidin™ and Aplidin analog-treated endothelial cells. Aplidin™ and analogs downregulated VASH1 and KDR protein levels after 24 h. **f** DKK-3 release from endothelial cells was reduced upon treatment with bortezomib, Aplidin™ or PM01215 and PM02781 after 72 h, as determined by a sandwich ELISA specific for human DKK-3. The means of one treatment group were compared to untreated control. The level of significance for the analysis was set at *p* < 0.05

Beside the analysis of oxidative stress and senescence we wanted to analyze effects of Aplidin and its analogs on induction of endoplasmatic reticulum (ER) stress and activation of unfolded protein response (UPR). Therefore we compared UPR responses in direct comparison to 10 nM thapsigargin, a strong inducer of UPR on human endothelial cells. Three different branches of UPR, the master regulator GRP78/HSP5A, the IRE1α/XPB1 s and PERK/CHOP/DDIT3 pathway were analyzed on gene expression level. Bortezomib, Aplidin and analogs did not induce HSP5A gene expression and splicing of XPB1 within 24 h (Fig. 5a/b). Only thapsigargin was able to increase GRP78 and XPB1 s protein as analyzed by Western Blot after 72 h (Fig. 5c). DDIT3/CHOP was significantly elevated on gene expression in bortezomib, Aplidin and analog-treated cells, but not in such a massive response as in thapsigargin-treated control cells (Fig. 5d).

### PM01215 and PM02781 decrease vascular maturation factors Vasohibin-1 and Dickkopf-3

Vascular maturation processes support mural cell coverage as well as capillary tube formation and are controlled by paracrine factors released by endothelial cells [18-21]. Therefore, we analyzed whether Aplidin analogs affect expression and release of Vasohibin-1 (VEGF target gene) and Dickkopf-3 protein. While bortezomib (5 nM) did not significantly alter VASH1 expression in endothelial cells after 24 h (*n* = 3; Fig. 5e), *VASH1* gene expression was significantly reduced after stimulation with Aplidin™ (5 nM) and its analogs (10 nM each). We also detected a downregulation of KDR (VEGFR2) protein expression in endothelial cells 24 h after stimulation with bortezomib or Aplidin analogs (Fig. 5e). Moreover, all antiangiogenic drugs tested significantly reduced Dickkopf-3 release into culture medium 24 h after incubation (Fig. 5f).

### PM01215 and PM02781 inhibit capillary tube formation and angiogenic sprouting of human endothelial cells

**Fig. 6:**
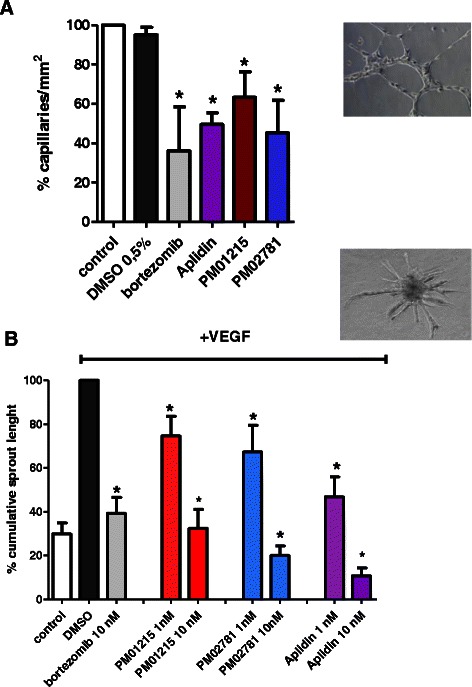
Analysis of capillary tube formation and angiogenic sprouting of human endothelial cells *in vitro*. **a** Capillary tube formation of bortezomib (5 nM) and Aplidin™ and derivatives (10 nM) -treated HUVECs was analyzed in triplicate over a time window of 6 h (*n* = 3, mean ± SEM of three different donors) in direct comparison to respective controls (0.1 % DMSO, bortezomib, Aplidin™). Untreated cells indicate 100 % capillaries/mm^2^ in matrigel. PM01215 and PM02781 significantly inhibited tube formation of HUVECs. The means of one treatment group are compared to untreated control. The level of significance for the analysis was set at *p* < 0.05. **b** Angiogenic sprouting of human endothelial cell spheroids in collagen type I/methylcellulose gel was analyzed after stimulation with 50 ng/mL human VEGF_165_. (*n* = 3, mean ± SEM of three different donors). DMSO-treated (0.1 %) cells with VEGF stimulation correspond to 100 % cumulative sprout length. All tested drugs significantly inhibited VEGF-induced sprouting at concentrations of 1 to 10 nM. The means of one treatment group were compared to the DMSO control. Stars indicate *p* values < 0.05

Antiangiogenic effects of the Aplidin derivatives were tested *in vitro* in functional 3D assays inducing capillary tube formation and generation of sprouts from endothelial spheroids. Tube formation of HUVECs (*n* = 3) in matrigel was inhibited by Aplidin™ (5 nM) and Aplidin derivatives (10 nM each, Fig. 6a). DMSO (0.1 %) served as negative control and bortezomib (5 nM) as positive control for inhibition of capillary tube formation.

In addition, HUVECs (*n* = 3) were cultured as spheroids in hanging drops for 24 h. Thereafter, spheroids were seeded into methylcellulose/collagen type I matrix together with 100 ng/mL VEGF and drugs (Fig. 6b). In comparison to 0.1 % DMSO (control) Aplidin™ and analogs significantly inhibited angiogenic sprouting at concentrations of 1 and 10 nM.

### PM01215 and PM02781 inhibit neovascularization and VEGF-induced angiogenesis in the chicken chorioallantoic membrane *in vivo*

**Fig. 7:**
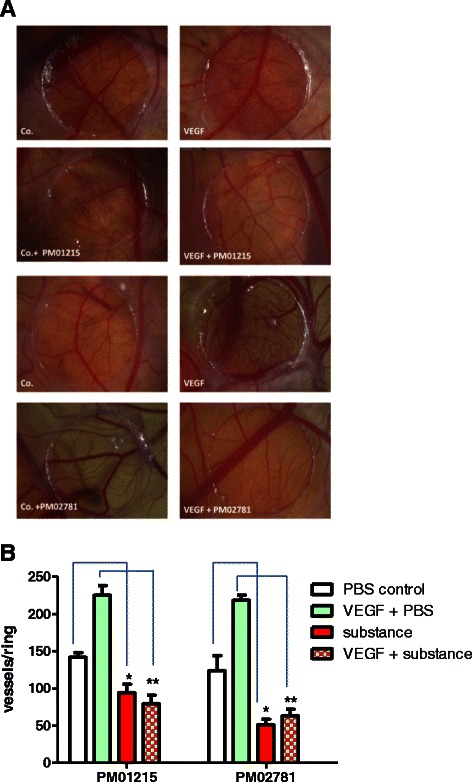
Aplidin analogs inhibit physiological and VEGF-induced angiogenesis in the chicken chorioallantoic membrane (CAM) *in vivo*. **a** Antiangiogenic effects of Aplidin derivatives were tested *in vivo* in CAM after repeated (2 x) topical application of 1 nmol of the drugs to Permanox™ rings with or without recombinant human VEGF_165_ (1 μg/ring). Representative CAM areas of chicken embryos were treated with Aplidin analogs and in combination with VEGF. **b** Statistical analysis for each compound (*n* = 6 animals). Aplidin analogs inhibited VEGF-induced angiogenesis (**, *p* < 0.05, comparison to human VEGF_165_ +  PBS) and physiological neovascularization (*, *p* < 0.05, comparison to PBS treatment)

Antiangiogenic effects of Aplidin derivatives were also tested *in vivo* in CAM after repeated topical application of 1 nmol of the drugs to Permanox™ rings with or without recombinant human VEGF (1 μg/ring; Fig. 7a). As expected, VEGF induced a strong angiogenic reaction that was efficiently blocked by simultaneous application of both Aplidin analogs (*n* = 6 for each group of animals, Fig. 7b). Moreover, we observed significant inhibition of spontaneous neovascularization in CAM (Fig. 7b). These data indicate that both analogs have antiangiogenic activities at sublethal concentrations in chicken embryos.

### PM01215 and PM02781 inhibit growth and vascularization of human multiple myeloma grafts in the chicken CAM

In addition, PM01215 and PM02781 were tested in a human multiple myeloma xenograft model in the chicken. The human myeloma cells OPM-2^eGFP^ were grafted together with human mesenchymal cells and collagen-type-I matrix on the CAM of chicken embryos.

**Fig. 8:**
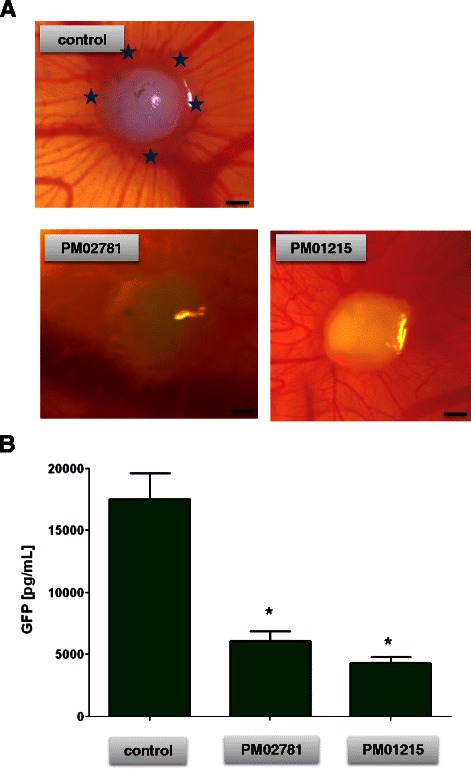
Aplidin analogs inhibit vascularization and growth of human myeloma xenografts in the chicken chorioallantoic membrane (CAM) *in vivo*. **a** Human multiple myeloma cell lines (OPM-2) were lentivirally transfected and selected to express stably green fluorescent protein (GFP). Then cells were mixed with primary human bone-marrow mesenchymal cells, collagen type-I as extracellular matrix component and with 1 nmol of Aplidin analogs PM01215 and PM02781. Spheroids (*n* = 6) were grafted on the chorioallantoic membrane of chicken embryos. Xenografts formed nice vascularized tumors after 4 days; PM02781 and PM01215 treated xenografts were less vascularized. Arrows indicate blood vessel sprouting close to tumor xenograft, bars indicate 500 μm. **b** Single MM xenografts were excised and homogenized in lysis puffer and thereafter, measured by ELISA. GFP concentrations of single tumors were calculated (*n* = 6, mean ± SEM; stars indicate p values <0.05, differences between the means of one treatment group and untreated control were analyzed). PM02781 and PM01215 treated myeloma xenografts displayed less tumor cell mass

Both, PM01215 and PM02781, significantly inhibited blood vessel formation adjacent to tumor grafts 5 days after incubation (Fig. 8a). Tumor cell mass was quantified by measuring the transgene GFP in myeloma cells in an ELISA after homogenization of tumors with adjacent host tissue. In comparison to control tumors, PM01215 and PM02781 treated grafts displayed significantly less tumor mass and cell growth (Fig. 8b). This strong reduction of tumor growth was also due to apoptosis of OPM-2 cells in the graft at these high concentrations (1 nmol). OPM-2 myeloma cells undergo apoptosis at high concentrations of Aplidin and analogs (Additional file 2: Table S2).

## Discussion

Tumor development and progression strongly depend on angiogenesis [2, 3]. Thus, inhibition of angiogenesis by “antiangiogenic drugs” represents an important tool for holding tumors in a small avascular state and inhibiting their growth and metastasis [5, 7]. Despite extensive research only few drugs primarily targeting “VEGF signaling” have reached clinical practice and currently face new challenges such as the development of resistances [7, 8, 22]. Therefore, there is an urgent need for novel compounds that act “antiangiogenically” by stopping endothelial cell proliferation without inducing apoptosis in the vascular network of the body and/or affecting coagulation processes. Next to the proteasome inhibitor bortezomib [13], the cyclodepsipeptide Aplidin™ originally isolated from the Mediterranean tunicate Aplidium albicans, has been demonstrated to exert antiangiogenic effects *in vitro* and *in vivo* [12]. This study identified two more easy to synthesize Aplidin analogs as potent antiangiogenic drugs, which in the low nM range induced cell cycle arrest in mitotic endothelial cells. Both analogs were less effective in the induction of apoptosis than the original Aplidin when used at same low nM concentrations. The lower toxicity might result by diminished uptake into human cells due to modification of side chains.

Both analogs induced cell cycle arrest in G1 phase and induced expression of the cyclin-dependent kinase inhibitor p16^INK4A^. Induction of p16^INK4A^ and senescence-associated beta galactosidase is one of the hallmarks of premature senescence and terminal growth arrest. Indeed, it was recently demonstrated by Jenkins et al. that oxidative stress, in particular radical oxygen species, induce p16^INK4A^ and arrest cells in G1 [23]. Nevertheless, we cannot provide the proof that induction of p16^INK4A^ is the main trigger for the observed terminal growth arrest or if there are still other mechanisms.

Further analyses revealed that Aplidin analogs induced oxidative stress in endothelial cells. Induction of oxidative stress has already been observed in breast and ovarian cancer cell lines after treatment with Aplidin™ [10, 24]. In comparison to these studies performed on tumor cells with high concentrations of Aplidin™ (400 nM), we were not able to rescue cells after adding antioxidants like N-acetyl-cysteine, although we used by far lower nM concentrations of Aplidin analogs. Primary endothelial cells remained in terminal growth arrest and could not be rescued by mitogenic growth medium for further proliferation. Our observations indicate that the cellular senescence program is activated by both Aplidin analogs PM01215 and PM02781.

With regard to changes in endothelial cells after treatment with PM01215 and PM02781 we observed alterations in vascular maturation factors. Release of the Dickkopf Homolog 3 (DKK-3) was downregulated after treatment with Aplidin analogs. DKK-3 has been shown to act on endothelial cells as a differentiation factor [20, 21] by inhibiting TGF-beta/Smad signaling [25] and supporting or regulating Wnt/beta-catenin activity [26]. Furthermore, we observed downregulation of the VEGF target gene *VASH1*. The encoded vasohibin protein has been shown to induce vascular maturation by supporting coverage of blood vessels with smooth muscle cells and pericytes [18, 19]. Noteworthy, it was recently shown that downregulation of vasohibin induces oxidative stress and premature senescence in human endothelial cells [27]. Thus, Aplidin analogs could enhance oxidative stress and senescence processes by downregulating vasohibin.

In particular, in our chicken multiple myeloma xenograft models we were able to demonstrate potent antiangiogenic and antimyeloma activities of both Aplidin analogs in sublethal concentrations. Our results of the novel Aplidin analogs are in line with the data of Cers et al. demonstrating the antiangiogenic and anti-myeloma activities of the original Aplidin™ in the 5TMM syngeneic model of multiple myeloma [9].

## Conclusion

Our data give evidence that both novel Aplidin analogs show potent antiangiogenic activities *in vitro* and *in vivo* assays at low nanomolar concentrations. Therefore, PM01215 and PM02781 are attractive candidates for the development of new antiangiogenic cancer drugs and warrant further analysis in mouse tumor models to study effects on tumor growth and blood vessel formation.

### Ethical standards

According to the Austrian law no local ethical approval is required for commercially available human primary cells. According to the Office of Laboratory Animal Welfare of the US public health service avian embryos are not considered live vertebrate animals until hatching. The NIH Office of Laboratory Animal Welfare has provided written guidance in this area (http://www.grants.nih.gov/grants/olaw/references/ilar91.htm and NIH Publication No.: 06–4515). Residual blood samples from volunteers were used after obtaining written informed consent of healthy donors for scientific research projects. From the local ethic commission of the Medical University of Innsbruck (UN4012) we have a permission to use anonymized, voluntarily donated peripheral blood as controls. This is now stated in the ethics paragraph on page 18, line 458.

## Additional files

Additional file 1: Table S1. Characterization of human primary cells. (DOCX 14 kb)
Additional file 2: Table S2. Sensitivity of multiple myeloma cell line (OPM-2) to drugs. (DOCX 15 kb)

## Abbreviations

ASMA: Alpha smooth muscle cell actin
CAM: Chorioallantoic membrane
DMSO: Di-methyl sulfoxide
eGFP: Enhanced green fluorescent protein
ELISA: Enzyme-linked Immunosorbent assay
HDF: Human diploid fibroblasts from bone-marrow
HUVEC: Human umbilical vein endothelial cells
NAC: N-acetyl cysteine
PBS: Phosphate buffered saline
PBMNC: Peripheral blood mononuclear cells
RT-PCR: Reverse transcriptase-polymerase chain reaction
RNA: Ribonucleic acid
VASH: Vasohibin
DKK: Dickkopf
