# Supplementary material for: The Aplidin analogs PM01215 and PM02781 inhibit angiogenesis in vitro and in vivo
Source: BMC Cancer. 2015 Oct 19;15:738. doi: 10.1186/s12885-015-1729-4 (PMC4615365; doi:10.1186/s12885-015-1729-4)
Supplement: Additional file 1: Table S1. — Characterization of human primary cells. (DOCX 14 kb) [file 12885_2015_1729_MOESM1_ESM.docx]

**Supplementary table 1: Characterization of human primary cells**

Flow cytometric analysis of human primary cells, for each cell type three different donors were analyzed for lymphocyte/monocytes (CD14, CD45), endothelial (CD31, KDR) and epithelial (EpCAM) markers**.**

| Cell type | CD14 | CD45 | CD31 | KDR | EpCAM |
| --- | --- | --- | --- | --- | --- |
| HUVECs | Neg (3/3) | Neg (3/3) | Pos (3/3) | Pos (3/3) | Neg (3/3) |
| PBMNCs | Pos (3/3) | Pos (3/3) | Pos (3/3) | Neg (3/3) | Neg (3/3) |
| HDFs | Neg (3/3) | Neg (3/3) | Neg (3/3) | Neg (3/3) | Neg (3/3) |
