# Supplementary material for: The Aplidin analogs PM01215 and PM02781 inhibit angiogenesis in vitro and in vivo
Source: BMC Cancer. 2015 Oct 19;15:738. doi: 10.1186/s12885-015-1729-4 (PMC4615365; doi:10.1186/s12885-015-1729-4)
Supplement: Additional file 2: Table S2. — Sensitivity of multiple myeloma cell line (OPM-2) to drugs. (DOCX 15 kb) [file 12885_2015_1729_MOESM2_ESM.docx]

**Supplementary table 2: Sensitivity of multiple myeloma cell line (OPM-2) to drugs**

| **OPM-2 (n=3)** | **Untreated**  **10.6± 1.6** |  |  |  |  |
| --- | --- | --- | --- | --- | --- |
| **concentration [nM]** |  | **bortezomib** | **Aplidin™** | **PM01215** | **PM 02781** |
| **10** |  | 33.5 ± 2.7 | 16.4 ± 1.2 | 16.2 ± 1.1 | 16.7 ± 1.1 |
| **50** |  | 41.8 ± 3.8 | 27.3 ± 1.1 | 17.9 ± 1.3* | 19.7 ± 2.9* |
| **100** |  | 42.5 ± 1.2 | 38.2 ± 1.2 | 21.9 ± 1.8* | 28.3 ± 0.9* |
|  |  |  |  |  |  |

OPM-2 cells were analyzed in triplicates by the Annexin V Apoptosis Detection Kit (eBioscience) and measured on flow cytometer. Apoptotic cells were defined as Annexin V positive /7AAD negative. Aplidin was inducing significant more apoptosis than analogs PM01215 and PM 02781 at concentrations of 50 or 100nM 24 h after incubation.
